# Improving S-Curve Bias Through Joint Compensation of HPA and Filter Distortions

**DOI:** 10.3390/s26030981

**Published:** 2026-02-03

**Authors:** Longyu Chen, Yi Yang, Tulin Xiong, Lin Chen, Yuqi Liu

**Affiliations:** Southwest China Research Institute of Electronic Equipment, Chengdu 610036, China; 202320000249@std.uestc.edu.cn (L.C.); yangyi355@163.com (Y.Y.); xiongtulin@126.com (T.X.); boolechen@163.com (L.C.)

**Keywords:** navigation signal transmission channel, signal quality evaluation, predistortion, QRD-RLS algorithm, lookup table

## Abstract

**Highlights:**

**What are the main findings?**

- This study reveals that the predistortion filter breaks the constant envelope characteristic of the signal, resulting in nonlinear distortion after passing through the HPA.
- To address the distortion introduced by both the filter and the power amplifier in the navigation signal transmission chain, this study proposes a joint compensation method, which effectively reduces the SCB.

**What are the implications of the main findings?**

- This study effectively enhances navigation signal quality and provides strong support for improving the stability and reliability of high-precision positioning services.
- The proposed compensation method serves as a reference for optimizing the design of navigation satellite payloads and provides a technical foundation for the evolution of future navigation systems toward high-fidelity signal generation.

**Abstract:**

Navigation signals are simultaneously affected by nonlinear distortion from the high-power amplifier (HPA) and linear distortion from the filter in the navigation signal transmission channel, which reduce the signal quality and degrade the performance in high-precision positioning services. To address the limitation of traditional compensation methods under nonlinear conditions, this proposes a joint compensation approach. The approach first employs an iterative piecewise optimization method to design a predistortion filter to enhance the compensation ability for linear distortion. Then a QR-decomposition recursive least squares parameter extraction algorithm is used to extract the actual HPA model and construct a lookup table, enabling adaptive compensation of nonlinear distortion. With S-curve bias (SCB) as the performance evaluation index, the results show that this method can significantly reduce the SCB and effectively compensate for the distortion. The findings indicate that the proposed method improves navigation signal quality and provides reliable support for high-precision positioning services.

## 1. Introduction

As one of the four major global navigation satellite systems (GNSS), the BeiDou Navigation Satellite System (BDS) provides high-precision and highly reliable positioning, navigation, and timing (PNT) services to various users worldwide under all-weather and all-time conditions [1]. Navigation signals are the key and fundamental components that satellite navigation systems provide PNT services [2]. However, non-ideal transmission channels of navigation signals can cause distortions [3], thereby degrading signal quality. These distortions degrade the signal waveform and correlation properties, leading to leading to systematic errors in pseudorange measurements. In subsequent GNSS processing, such biases may cause failures in ambiguity resolution or introduce systematic errors, thereby degrading pseudorange positioning accuracy [4]. Advanced positioning techniques, such as direction-of-arrival (DOA)-based target localization, as well as related parameter estimation processes, rely on accurate signal models and waveform characteristics [5,6]. These studies highlight the critical importance of maintaining signal fidelity in high-precision positioning systems.

GNSS aims to provide higher-precision positioning services, which imposes more stringent requirements on signal quality, thus requiring more refined control of navigation signal quality. Therefore, it is essential to investigate the distortion mechanisms in the navigation signal transmission channel and to evaluate and analyze navigation signal quality, in order to further improve the performance of the navigation system.

The post-filter in the navigation signal transmission channel can introduce group delay jitter, which severely degrades signal quality. References [7,8] systematically analyzed the mechanism by which group delay affects signal quality. Reference [9] proposed a theoretical method for evaluating S-curve bias (SCB) based on channel and signal parameters, and analyzes the effect of second-order group delay on the SCB. Reference [10] further revealed that the phase differences between subcarriers caused by odd-order group delay are the main source of phase bias. Reference [11] proposed a scheme of predistortion filters to compensate for group delay distortion. Reference [12] designed a low-order finite impulse response (FIR) pre-distortion filter based on sparse representation. Reference [13] further proposed a segmented filter design method to optimize compensation performance. However, these studies mainly focus on compensation for the filter and ignore the influence of the high-power amplifier (HPA). To improve satellite energy efficiency, HPAs usually operate near saturation, exhibiting potential nonlinear effects. Reference [14] indicates that the pre-band limiting filter in the transmission channel inevitably destroys the constant-envelope characteristics of the signal, causing nonlinear distortion after passing through the HPA, thereby weakening the effectiveness of compensation for the filter. Therefore, to achieve higher-precision positioning services, it is necessary to consider compensating for the nonlinear distortion by the HPA.

Currently, common power amplifier (PA) linearization techniques mainly include output back-off, feedforward linearization, negative feedback, envelope elimination and restoration, linear amplification with nonlinear components, and predistortion techniques [15]. With the advantages of broad applicability, high stability, and excellent linearization performance, digital predistortion (DPD) has become the most widely used linearization technique in communication systems. DPD techniques can be generally categorized into two types: lookup table (LUT) and polynomial model-based approaches. Compared with polynomial models, the LUT-based method is simpler to implement and has lower computational complexity [16,17], making it more suitable for resource-constrained spaceborne systems. However, the LUT-based method is constrained by the relationship between lookup accuracy and the number of table entries. A large number of table entries are usually required to obtain higher compensation accuracy, which results in increased storage overhead. To reduce the storage overhead of LUTs, references [18,19] adopt linear interpolation to decrease the number of table entries while maintaining compensation accuracy. Existing PA linearization techniques are primarily applied in communication systems to suppress out-of-band spectral regrowth and improve error vector magnitude (EVM) performance. However, the improvement of navigation signal quality through PA predistortion, especially when cascaded with a predistortion filter, lacks in-depth research.

Current research on the overall distortion compensation for navigation signal transmission channels remains relatively limited. Existing studies focus primarily on compensating for filter distortion, and investigations of compensation techniques for non-ideal characteristics are almost exclusively conducted on a single PA or filter. Reference [20] analyzed the effects of the pre-filter and PA on the signal, but does not consider the distortion introduced by the post-filter. Reference [21] considered the compensation problem of both the post-filter and PA. It provided the unit impulse response of the predistortion filter, but does not further elaborate on its practical implementation.

To address the limitations of existing studies on joint compensation, this work systematically investigates the distortion in navigation signal transmission channels and proposes a joint compensation method for HPA and filters. This method employs an iterative piecewise optimization method to design the predistortion filter and utilizes a QR-decomposition recursive least squares (QRD-RLS) adaptive algorithm to model the actual HPA to construct a lookup table, thereby achieving joint predistortion compensation for both the filter and HPA. Simulation results show that the proposed method can effectively compensate for signal distortion and significantly improves signal quality.

## 2. Modeling of Navigation Signal Transmission Channels and Analysis of SCB

**Figure 1:**
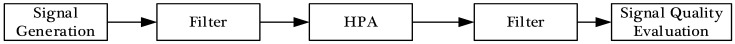
Equivalent baseband model of navigation signal transmission channel.

The equivalent baseband model of the navigation signal transmission channel is shown in Figure 1. The primary function of the pre-filter is to filter out image-frequency components and suppress out-of-band noise. Since it can be regarded as an ideal filter with minimal impact on the linear distortion, its influence on the signal is not considered in this work. The HPA is used to amplify the signal power, but when the input signal exhibits a non-constant envelope, the HPA introduces nonlinear distortion. The post-filter suppresses unwanted out-of-band spurious signals and high-order harmonics from the high-power signals, resulting in linear distortion.

SCB reflects the symmetry of the cross-correlation function (CCF). The code tracking loop utilizes the symmetry of the CCF to make the output of the code discriminator as close to 0 as possible, thereby aligning the code phase of the reproduced and received signals. Signal distortion affects the symmetry of the CCF, causing a deviation between the code phase of the receiver output and the actual phase of the received signal, resulting in pseudorange bias, which can be obtained by multiplying the SCB by the speed of light. Therefore, SCB is commonly used as a key indicator for evaluating navigation signal quality.

The Early-Mines-Late Power (EMLP) discriminator is a commonly used non-coherent code tracking discriminator, which estimates the code phase by comparing the power difference between the early and late correlator branches [22]. The discriminator curve of the EMLP code tracking discriminator [23,24] can be expressed as follows,

(1) $$D(\tau ,\delta )={\left|\mathrm{CCF}\left(s_{rec}(t),s_{ref}\left(t+\tau +\frac{\delta }{2}\right)\right)\right|}^{2}-{\left|\mathrm{CCF}\left(s_{rec}(t),s_{ref}\left(t+\tau -\frac{\delta }{2}\right)\right)\right|}^{2},$$

where $s_{rec}$ is the received signal, $s_{ref}$ is the local reference signal, δ represents the correlator interval, and $\mathrm{CCF}\left(s_{rec}(t),s_{ref}\left(t+\tau \right)\right)$ is the cross-correlation function between the received signal and the reference signal. Specifically, $\mathrm{CCF}\left(s_{rec}(t),s_{ref}\left(t+\tau +\frac{\delta }{2}\right)\right)$ and $\mathrm{CCF}\left(s_{rec}(t),s_{ref}\left(t+\tau -\frac{\delta }{2}\right)\right)$, respectively, represent the output of the early (E) and late (L) correlator branch. The code phase delay τ is obtained from the zero-crossing of the discriminator function D(τ,δ). For a certain correlator interval δ, $D\left(\tau_{b}(\delta ),\delta \right)=0$ at $\tau =\tau_{b}(\delta )$, and the CCF reaches its maximum value at $\tau =\tau_{0}$. Accordingly the SCB is equal to $\tau_{b}(\delta )-\tau_{0}$.

## 3. Predistortion Design for Navigation Signal Transmission Channels

**Figure 2:**
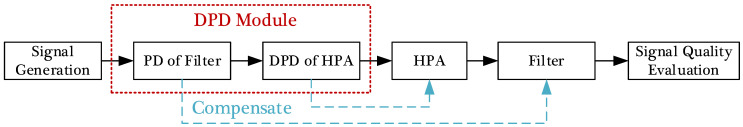
Joint predistortion baseband model. The dashed arrows represent compensation paths, where the PD of the filter and the DPD of the HPA compensate for the corresponding nonlinear modules.

Distortion in the navigation signal transmission channel primarily consists of two components: nonlinear distortion introduced by the HPA and linear distortion introduced by the filter. Although satellite downlink navigation signals are constant-envelope signals, applying a predistortion filter to compensate for the filter breaks the constant envelope characteristics. As a result, additional nonlinear distortion is introduced by the HPA, which degrades the compensation performance of the filter. To address this issue, this paper proposes a joint predistortion method that compensates for the HPA and the filter. The overall model is shown in Figure 2. The predistortion module consists of a predistortion filter and an HPA predistorter. The HPA predistorter is specifically designed to compensate for the HPA. By treating the cascaded predistortion module as an ideal channel, the predistortion filter can effectively compensate for the distortion introduced by the filter.

### 3.1. Design of an Iterative Piecewise Predistortion Filter

Linear distortion introduced by the filter consists of amplitude distortion and phase distortion. In the transmission channel, the amplitude distortion caused by the filter is relatively minor, whereas group delay is the primary factor affecting the quality of the navigation signal. Therefore, this paper does not consider amplitude distortion, assumes that the amplitude-frequency response within the band is always 1, and focuses solely on the effect of group delay. The design principle of the predistortion filter is as follows.

The definition of group delay [25,26] is as follows:

(2) $$grp(\omega )=-\frac{d\varphi (\omega )}{d\omega },$$

where φ(ω) represents the phase, ω=2∗pi∗f, and f is the corresponding frequency.

The channel group delay is estimated from the input and output signals, and then a polynomial model is used to fit the group delay. The model is shown below:

(3) $$G_{d}\left(f\right)=\sum_{k=0}^{K}\beta_{k}f^{k},$$

where $\beta_{k}(k=0,1,\ldots ,K)$ represents the coefficients of the model.

Based on Equation (3), the phase-frequency response of the filter can be obtained as follows:

(4) $$\phi (f)=-\sum_{k=0}^{K}\frac{1}{k+1}\beta_{k}f^{k+1}.$$

Accordingly, the frequency response of the predistortion filter is given by:

(5) $$H_{obj}(f)=e^{j2\pi \phi (f)}.$$

The coefficients of the predistortion filter can be obtained according to Equation (5). Assuming the designed filter group delay is $grp_{ori}$, the corresponding phase-frequency response $\varphi_{ori}$ can be obtained by integration, and the frequency response is given by

(6) $$H_{ori}=e^{j\varphi_{ori}}.$$

However, the compensation accuracy of the filter designed based solely on this approach is limited. In order to improve the compensation performance, we propose an iterative piecewise predistortion method to optimize the filter parameters and enhance the compensation accuracy.

First, the frequency band is divided into segments, and then calculate the frequency-response error between the designed predistortion filter and the target predistortion filter in each frequency band,

(7) $$H_{error}(f(n))=H_{obj}(f(n))-H_{ori}(f(n)).$$

The error function for each frequency band is obtained by multiplying $H_{error}(f(n))$ by the corresponding weight w(n),

(8) $$H_{error}=[\begin{array}{ccc} H_{error}(f(1))\times w(1) & \cdots & H_{error}(f(N))\times w(N) \end{array}].$$

The problem of solving the filter coefficients can thus be transformed into an optimization problem, that is, solving

(9) $$\min\left|H_{error}\right|.$$

By solving this optimization problem, the optimized filter coefficients and the corresponding group delay are obtained. However, this group delay still differs from the target group delay. Therefore, the required adjustment to the group delay is calculated, which is the difference between the designed filter group delay $grp_{opt}$ and the target group delay $grp_{obj}$.

(10) $$grp_{error}=grp_{obj}-grp_{opt}.$$

Update the group delay objective function,

(11) $$grp_{new\_obj}=grp_{obj}+grp_{error},$$

and then obtain the new filter frequency response,

(12) $$H_{new\_obj}=e^{j\varphi_{new\_obj}}.$$

The new coefficients of the predistortion filter are obtained according to Equation (12). Repeat the above steps until the group-delay error of the designed filter is less than the predefined threshold or the maximum number of iterations is reached. Finally, the iteratively optimized filter coefficients are obtained.

### 3.2. DPD for the HPA

In the ideal case, the output signal of the HPA is linearly amplified from the input signal. When the input signal is small, the HPA operates in the linear amplification region, and the input signal is linearly amplified. However, to improve efficiency, HPA is typically driven into the saturation region, which results in severe nonlinear distortion. Such nonlinear distortion results in both in-band distortion and out-of-band spectral regrowth. In-band distortion affects the amplitude and phase of the signal, thereby degrading signal quality, while out-of-band spectral regrowth refers to expansion of the signal bandwidth, which interferes with adjacent channels, and ultimately degrades system performance.

#### 3.2.1. Saleh Model and the QRD-RLS Algorithm

Adel A. M. Saleh first proposed the Saleh model [27], which is commonly employed as an HPA in satellite onboard systems. The Saleh model employs two functions to separately describe the amplitude distortion and phase distortion introduced by the HPA. The mathematical expressions are as follows:

(13) $$A(r)=\frac{\alpha_{a}r}{1+\beta_{a}r^{2}},$$

(14) $$\varphi (r)=\frac{\alpha_{\phi }r^{2}}{1+\beta_{\phi }r^{2}},$$

where A(r) denotes the amplitude transfer function, φ(r) denotes the phase transfer function, r represents the input signal amplitude, and $\alpha_{a}$, $\beta_{a}$, $\alpha_{\varphi }$, and $\beta_{\varphi }$ are model parameters.

In the simulations, the HPA is modeled using a parameter estimation algorithm based on the actual characteristics. Although the least squares (LS) algorithm is one of the most widely used parameter estimation algorithms, it requires a high computational cost to invert the correlation matrix of the data matrix. By contrast, QR decomposition can avoid the matrix inversion operation, reduce computational complexity and improve the stability of the algorithm. Therefore, this paper adopts the QRD-RLS adaptive algorithm to estimate the HPA model parameters, in order to reduce computational complexity and save resources.

First, rewrite Equations (13) and (14) as

(15) $$W_{A}=\frac{r}{A\left(r\right)}=\frac{1}{\alpha_{a}}+\frac{\beta_{a}}{\alpha_{a}}r^{2}=H\theta_{A},$$

and

(16) $$W_{\varphi }=\frac{r^{2}}{\varphi \left(r\right)}=\frac{1}{\alpha_{\varphi }}+\frac{\beta_{\varphi }}{\alpha_{\varphi }}r^{2}=H\theta_{\varphi },$$

where $\theta_{A}={\left[\begin{array}{cc} \frac{1}{\alpha_{a}} & \frac{\beta_{a}}{\alpha_{a}} \end{array}\right]}^{T}$, $\theta_{A}={\left[\begin{array}{cc} \frac{1}{\alpha_{\varphi }} & \frac{\beta_{\varphi }}{\alpha_{\varphi }} \end{array}\right]}^{T}$ and $H={\left[\begin{array}{cc} I & r^{2} \end{array}\right]}_{N*2}$.

According to the LS algorithm [28], we can obtain

(17) $$\theta_{A}={\left(H^{H}H\right)}^{-1}HW_{A},$$

and

(18) $$\theta_{\varphi }={\left(H^{H}H\right)}^{-1}HW_{\varphi }.$$

The following section introduces the QRD-RLS algorithm based on the Saleh model for estimating $\theta_{A}$. The estimation of $\theta_{\varphi }$ can be obtained in the same method.

Multiplying both sides of Equation (17) by $H^{H}H$, we obtain

(19) $$H^{H}H\theta_{A}=H^{H}W_{A}.$$

For the matrix H, when the length of the observation data is greater than the number of coefficients to be estimated, i.e., when N>2, there exists an N-dimensional orthogonal matrix Q such that

(20) $$QH=\left[\begin{array}{c} R\\ 0 \end{array}\right],$$

where R is a two-dimensional upper triangular matrix, and 0 is the zero matrix of $\left(N-2\right)*2$. Since Q is an orthogonal matrix, $Q^{H}Q=I$. Combined with Equation (20), Equation (19) can be rewritten as

(21) $$\left[\begin{array}{c} R^{H}\\ 0 \end{array}\right]Q^{H}Q\left[\begin{array}{c} R\\ 0 \end{array}\right]\theta_{A}=\left[\begin{array}{c} R^{H}\\ 0 \end{array}\right]QW_{A}.$$

By block-partitioning the matrix $QW_{A}$ as ${\left[z^{T},b^{T}\right]}^{T}$, where $z\in C^{2}$ and $b\in C^{N-2}$, we obtain

(22) $$R\theta_{A}=z.$$

To solve for $\theta_{A}$, we only need to know R and z.

The QR decomposition of a matrix can be accomplished using Givens rotation. Givens rotation can eliminate an element in the matrix, setting it to 0.

For a two-dimensional vector $v={\left[v_{1},v_{2}\right]}^{T}$, there exists an orthogonal matrix G, making $Gv={\left[{\left\|v\right\|}^{2},0\right]}^{T}$. The corresponding orthogonal matrix is

(23) $$G=\left[\begin{array}{cc} c & s\\ -s^{*} & c^{*} \end{array}\right],$$

where $c=\frac{v_{1}^{*}}{\sqrt{v_{1}^{2}+v_{2}^{2}}}$ and $s=\frac{v_{2}^{*}}{\sqrt{v_{1}^{2}+v_{2}^{2}}}$ [29].

Assuming that the QR decomposition of the data matrix at time n has been completed, we obtained

(24) $$Q(n)H(n)=\left[\begin{array}{c} R(n)\\ 0 \end{array}\right],$$

and

(25) $$Q(n)W_{A}(n)=\left[\begin{array}{c} \hat{z}(n)\\ \hat{z}’(n) \end{array}\right].$$

Since H and $W_{A}$ are the same transformation, we can combine them into an augmented matrix $\bar{\Phi }$, such that the transformation matrix acts on both simultaneously. Equations (24) and (25) can be rewritten as

(26) $$Q(n)\bar{\Phi }(n)=\bar{R}(n)=\left[\begin{array}{cc} R(n) & \hat{z}(n)\\ 0 & {\hat{z}}^{\prime }(n) \end{array}\right].$$

To obtain $R\left(n+1\right)$ at time n+1, we need to perform QR decomposition on. $\bar{R}(n+1)$. That is, QR decomposition is performed on

(27) $$\bar{R}(n+1)=\left[\begin{array}{cc} \sqrt{\lambda }R(n) & \sqrt{\lambda }\hat{z}(n)\\ h^{n+1}(n+1) & z(n+1) \end{array}\right],$$

where $h^{n+1}(n+1)=H(n+1,:)$ and z(n+1)=y(n+1). To eliminate $h^{n+1}(n+1)$ in $\bar{R}(n+1)$, first eliminate the element in the first column of the last row of $\bar{R}(n+1)$.

(28) $$\begin{array}{c} G_{1}(n+1)\bar{R}(n+1)=\left[\begin{array}{ccc} c_{1} & 0 & s_{1}\\ 0 & I & 0\\ -s_{1}^{*} & 0 & c_{1}^{*} \end{array}\right]\times \left[\begin{array}{ccc} \sqrt{\lambda }r_{1,1}(n) & \sqrt{\lambda }r_{1,2}(n) & \sqrt{\lambda }{\hat{z}}_{1}(n)\\ \vdots & \vdots & \vdots \\ h_{1}^{\left(1\right)}\left(n+1\right) & h_{2}^{\left(1\right)}\left(n+1\right) & z^{\left(1\right)}(n+1) \end{array}\right]\\ =\left[\begin{array}{ccc} r_{1,1}(n+1) & r_{1,2}(n+1) & {\hat{z}}_{1}(n+1)\\ 0 & \vdots & \vdots \\ 0 & h_{2}^{\left(2\right)}\left(n+1\right) & z^{\left(2\right)}(n+1) \end{array}\right] \end{array},$$

where $c_{1}=\frac{\sqrt{\lambda }r_{1,1}{\left(n\right)}^{*}}{\sqrt{|\sqrt{\lambda }r_{1,1}(n){|}^{2}+|h_{1}^{\left(1\right)}(n+1){|}^{2}}}$ and $s_{1}=\frac{h_{1}^{\left(1\right)}{\left(n+1\right)}^{*}}{\sqrt{|\sqrt{\lambda }r_{1,1}(n){|}^{2}+|h_{1}^{\left(1\right)}(n+1){|}^{2}}}$. Then, set the element in the second column of the last row to zero. The corresponding transformations on the two rows are as follows:

(29) $$\left[\begin{array}{cc} c_{2} & s_{2}\\ -s_{2}^{*} & c_{2}^{*} \end{array}\right]\times \left[\begin{array}{ccc} 0 & \sqrt{\lambda }r_{2,2}(n) & \sqrt{\lambda }{\hat{z}}_{2}(n)\\ 0 & h_{2}^{\left(2\right)}(n+1) & z^{\left(2\right)}(n+1) \end{array}\right]=\left[\begin{array}{ccc} 0 & r_{2,2}(n+1) & {\hat{z}}_{2}(n+1)\\ 0 & 0 & z^{\left(3\right)}(n+1) \end{array}\right],$$

where $c_{2}=\frac{\sqrt{\lambda }r_{2,2}{\left(n\right)}^{*}}{\sqrt{|\sqrt{\lambda }r_{2,2}(n){|}^{2}+|h_{2}^{\left(2\right)}(n+1){|}^{2}}}$ and $s_{2}=\frac{h_{2}^{\left(2\right)}{\left(n+1\right)}^{*}}{\sqrt{|\sqrt{\lambda }r_{2,2}(n){|}^{2}+|h_{2}^{\left(2\right)}(n+1){|}^{2}}}$. After two Givens rotations, all of the elements of $h^{n+1}(n+1)$ in $\bar{R}(n+1)$ are eliminated.

Thus, R and z can be obtained, and $\theta_{A}$ can be solved according to Equation (22). Since R is an upper triangular matrix, the solution for $\theta_{A}$ starts from the last row and then recursively calculates the other elements from bottom to top. This allows the model coefficients to be computed easily, avoiding the matrix inversion operation.

#### 3.2.2. Principle of the LUT

Due to the limited onboard computing resources, LUT is more widely used in satellite systems because of its simple structure and low computational cost. We construct a predistortion LUT based on the HPA model obtained in Section 3.2.1. The amplitude LUT stores the amplitudes of the HPA input and output signals, with the output signal amplitude as the index address and the input signal amplitude as the LUT output. The phase LUT stores the input signal amplitude and the negative value of the output signal phase distortion, with the input signal amplitude as the index address and the phase-compensation value as the LUT output.

**Figure 3:**
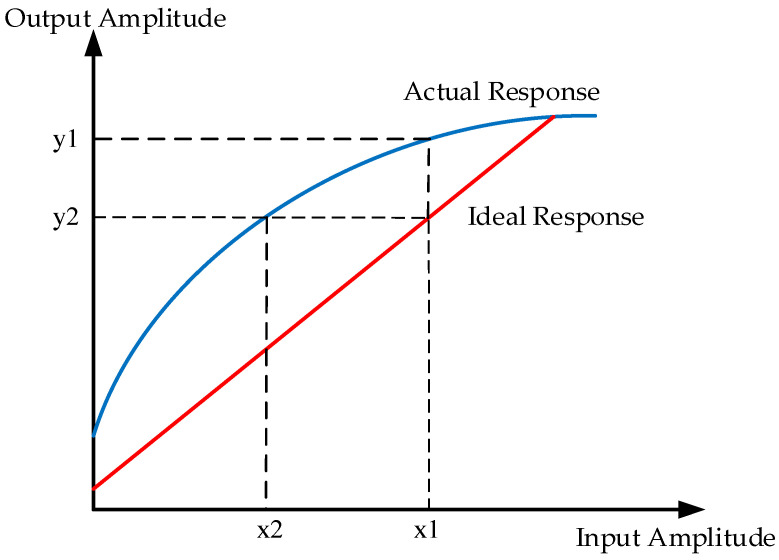
Principle of the lookup table.

The principle of the LUT is shown in Figure 3. Assume the amplitude of the input signal is $x_{1}$, and the actual HPA output amplitude is $y_{1}$, but the ideal output amplitude is $y_{2}$. Based on the actual amplitude modulation to amplitude modulation (AM-AM) characteristics of the HPA, the amplitude LUT output $x_{2}$ corresponding to $y_{2}$ is obtained. Utilizing $x_{2}$ to index the phase LUT, the corresponding phase-compensation output can be retrieved.

In a uniformly quantized LUT structure, when the quantization interval is too wide, the amplitude of the input signal may fall between two adjacent index addresses. In this case, the predistortion compensation value stored in the LUT can differ significantly from the actual compensation required by the signal, resulting in degraded predistortion performance. To achieve better performance while reducing the number of LUT entries, linear interpolation can be applied. An additional field can be added to the LUT to store the slope between adjacent entries thereby obtaining more accurate compensation.

### 3.3. Computational Complexity Analysis

To evaluate the engineering feasibility of the proposed joint compensation method on a spaceborne satellite platform, this subsection analyzes the computational complexity of a single execution of the algorithm and focuses on the computational overhead of the key processing steps, including the predistortion filter design, QRD-RLS parameter estimation and the construction of the LUT.

During the predistortion filter design, the group delay of the transmission channel is first calculated and then fitted using a polynomial model. The number of frequency-domain sampling points is denoted as $N_{f}$ and the order of the polynomial fitting is denoted as K. The computational complexities of the group delay calculation and the polynomial fitting are $O(N_{f})$ and $O(N_{f}*K^{2})$, respectively. Based on the fitted group delay model, the initial target frequency response of the predistortion filter $H_{obj}(f)$ is obtained, whose computational complexity is also $O(N_{f})$. Since K is typically chosen as a small value (set to K=3 in this work), the overall computational complexity of computing the initial predistortion filter frequency response can be approximated as $O(N_{f})$.

During the subsequent iterative optimization process, the order of the designed FIR predistortion filter is denoted as M. When a filter design method based on the least squares criterion is adopted, the corresponding computational complexity can be expressed as $O(N_{f}*M^{2})$. The frequency response of the filter is then computed based on the filter coefficients, with a computational complexity of $O(N_{f}*\log N_{f})$. Subsequently, the piecewise weighted error between the frequency response of the designed predistortion filter and the target frequency response is evaluated, with a computational complexity of $O(N_{f})$. Based on this error function, the filter coefficients are optimized using the least squares method, resulting in a computational complexity of $O(N_{f}*M^{2})$. Finally, the group delay of the filter is calculated from the optimized filter coefficients, and the target frequency response is updated, with a computational complexity of $O(N_{f}*\log N_{f})$.

The above procedures constitute the main operations performed in a single iteration. Assuming the number of iterations is I, the total computational complexity of the predistortion filter design process can be expressed as $O(I*(N_{f}*M^{2}+N_{f}*\log N_{f}))$.

It can be observed that the computational complexity of the proposed method mainly increases with the number of frequency-domain sampling points $N_{f}$, the filter order M, and the number of iterations I. It should be noted that the above complexity analysis corresponds to a single filter parameter optimization process. In practical applications, the predistortion filter parameters do not need to be updated frequently. Therefore, the computational overhead is acceptable in engineering practice.

When the QRD-RLS algorithm is employed to estimate the coefficients of the Saleh power amplifier model, the number of samples is denoted as $N_{s}$, and the number of parameters to be estimated in the amplitude distortion model is denoted as P. During each construction of the augmented matrix $\bar{R}(n+1)$, the computational complexity is $O\left(P\right)$. Subsequently, the augmented matrix $\bar{R}(n+1)$ is processed using Givens rotations. Each rotation requires updating $P\left(P+1\right)$ elements, and P rotations are performed for each sample. Therefore, the computational complexity for a single sample is $O\left(P^{2}\right)$. Considering all $N_{s}$ samples, the overall computational complexity of the amplitude distortion model parameter estimation is $O\left(N_{s}*P^{2}\right)$.

The parameter estimation process for the phase distortion model is exactly the same as that for the amplitude distortion model, and its computational complexity is also $O\left(N_{s}*P^{2}\right)$. In fact, the numbers of parameters in both the amplitude distortion and phase distortion models of the Saleh model P are 2. Therefore, the computational complexity of this parameter estimation process is relatively low, mainly increasing linearly with the number of samples $N_{s}$. It should be noted that the QRD-RLS parameter estimation can be performed offline and therefore does not impose additional computational burden on the onboard real-timing processing.

For the construction of the LUT, assume the LUT contains N entries. Based on the established HPA model, the corresponding input–output amplitude and phase mapping relationships can be directly generated and stored in the LUT. Subsequently, only N−1 difference operations between adjacent entries are required to compute the slopes for linear interpolation, thereby completing the LUT construction. Therefore, the computational complexity of the LUT construction process is O(N). From the above analysis, the LUT construction involves only simple mapping and difference operations. Its computational complexity increases linearly with the number of entries N, and the computational overhead is extremely low, making it suitable for implementation on a spaceborne satellite platform with limited computing resources.

In summary, the proposed joint compensation method exhibits well-controlled computational complexity across all processing stages. The computational complexity of predistortion filter design is mainly affected by iterative frequency-domain processing; the computational complexity of HPA model parameter estimation based on QRD-RLS increases linearly with data length and can be carried out offline; and the LUT construction process only involves simple arithmetic operations. Overall, the above complexity analysis indicates that the proposed joint compensation method can satisfy the computational resource constraints and engineering feasibility requirements of spaceborne platforms.

## 4. Experimental Validation and Results Analysis

### 4.1. Performance Verification of Predistortion Filter

**Figure 4:**
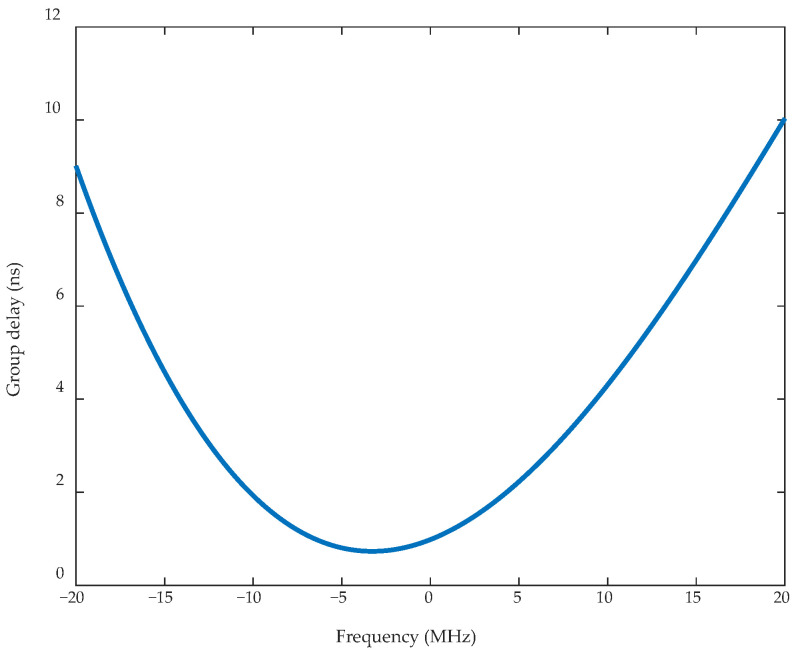
Group delay of the post-filter.

**Figure 5:**
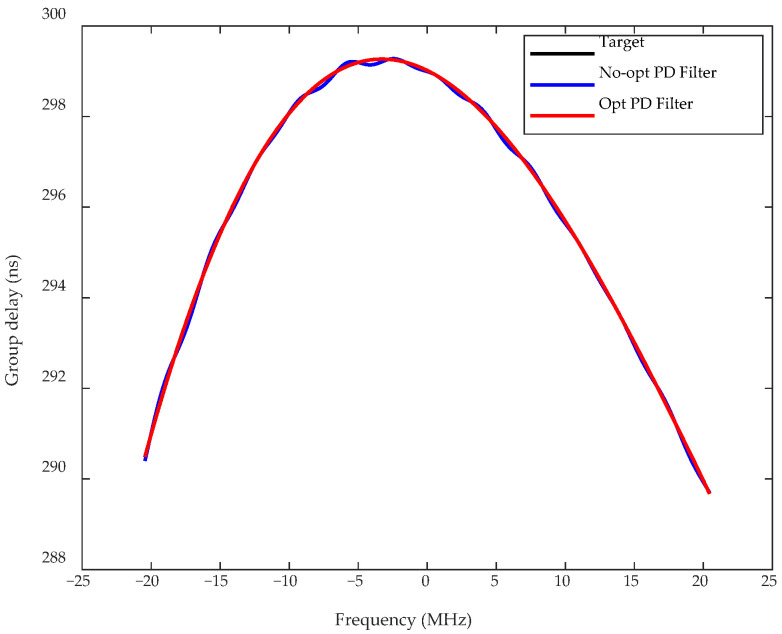
Group delay before and after optimization of the predistortion filter.

A set of measured filter group delays is shown in Figure 4. Based on this filter model, the predistortion filter is designed using the filter optimization method described in Section 3.1. Figure 5 shows the group delay of the predistortion filters designed using the proposed optimization method and the non-optimized method. Table 1 presents the group delay errors before and after optimization. Compared with the target predistortion filter, the maximum in-band group delay error of the designed predistortion filter is reduced from the order of 10^−1^ ns to 10^−3^ ns.

### 4.2. Performance Evaluation of the QRD-RLS Algorithm

**Figure 6:**
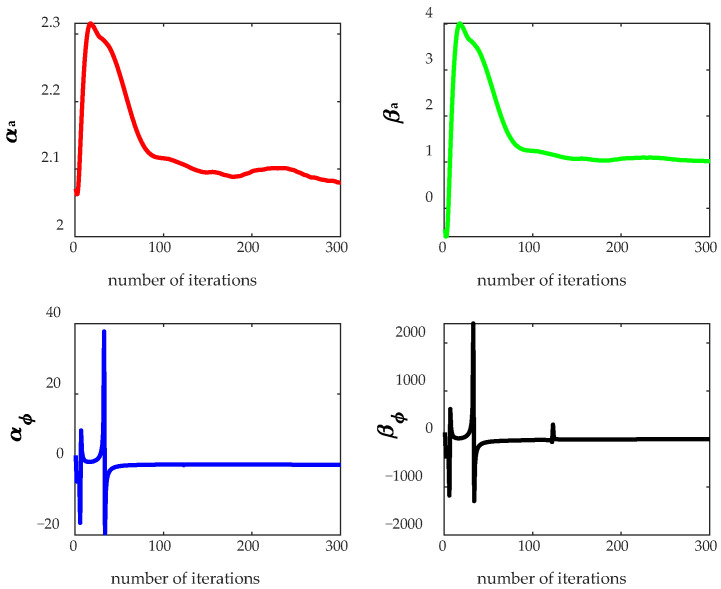
Performance of the QRD-RLS algorithm.

The TWTA model was extracted using the QRD-RLS parameter estimation algorithm described in Section 3.2.1. Figure 6 shows the estimation results of the Saleh model parameters under different iteration numbers. It can be observed that the estimation results gradually converge with the increase of the iteration number, and tend to stabilize when the iteration number is approximately 100.

### 4.3. Verification and Analysis of the Joint Compensation Method

**Figure 7:**
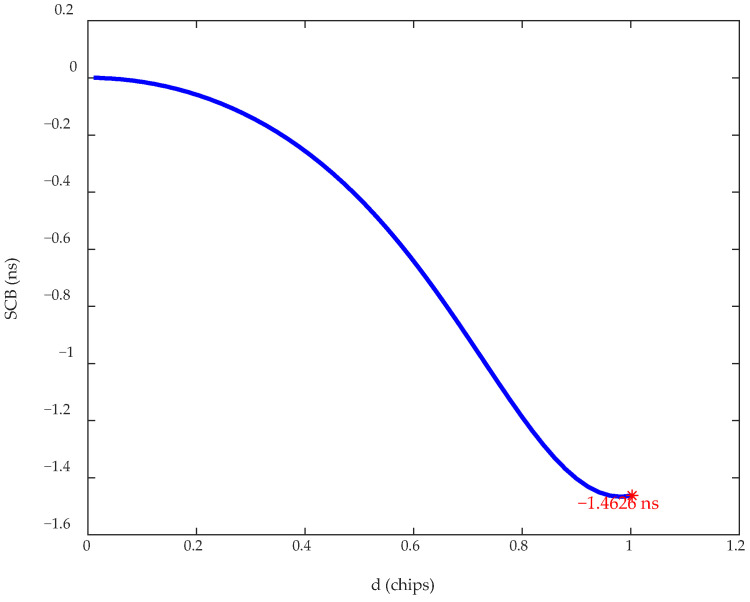
Impact of navigation signal transmission channel distortion on SCB. * marks the maximum value of the corresponding curve.

The BPSK baseband signal is used for simulation, with a code rate of Rc=10.23 Mcps. The SCB of the signal after passing through the HPA and the filter is shown in Figure 7. The results indicate that the signal undergoes distortion in the transmission channel, which degrades the SCB to 1.4626 ns.

**Figure 8:**
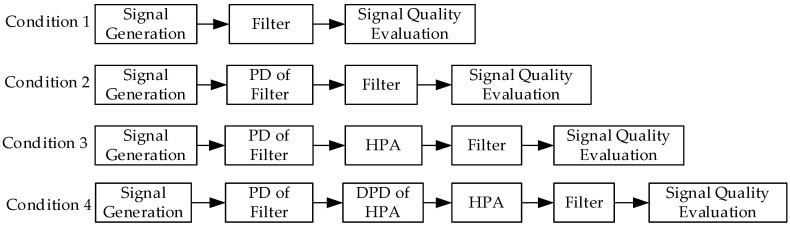
Simulation conditions.

**Figure 9:**
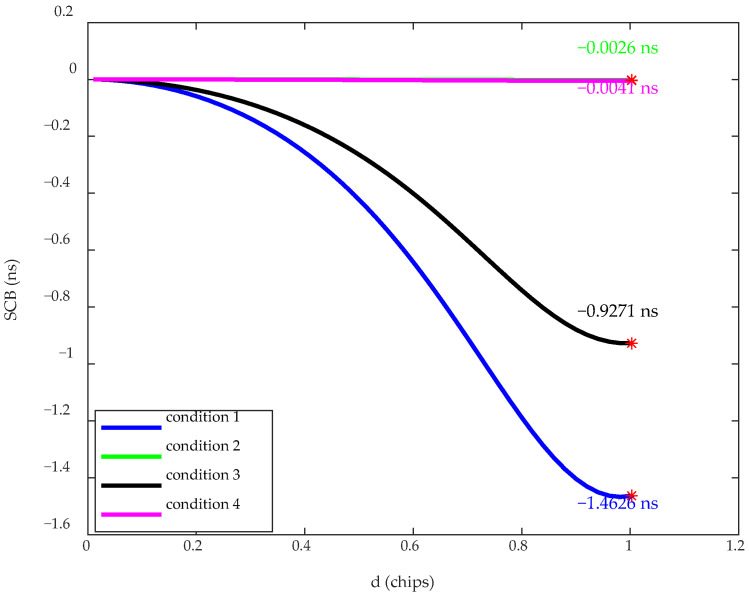
SCB simulation results of the proposed method under different simulation conditions. * marks the maximum value of the corresponding curve.

To verify the impact of the distortion introduced by the HPA and the filter on the SCB, as well as the performance of the corresponding predistortion compensation, simulations were conducted under four different conditions. The models for each test condition are shown in Figure 8, and the simulation results are presented in Figure 9.

Comparing the results of Figure 7 with the results of Condition 1 in Figure 9 verifies that a constant envelope signal does not undergo distortion when passing through the HPA. The results of Conditions 1 and 2 show that the predistortion filter designed in this paper achieves the intended performance, reducing the SCB to 0.0026 ns. In Condition 3, when the HPA is taken into account, the SCB deteriorates to 0.9271 ns. The comparison between Conditions 2 and 3 confirms that the predistortion filter breaks the constant envelope characteristic of the signal, causing nonlinear distortion after passing through the HPA, thus degrading the filter compensation performance. The results of Condition 4 show that, after applying the DPD of the HPA, the SCB decreases to 0.0041 ns, and the overall compensation for distortion in the navigation signal transmission channel achieves the intended performance.

### 4.4. Experimental Validation of the Joint Compensation Method

**Figure 10:**
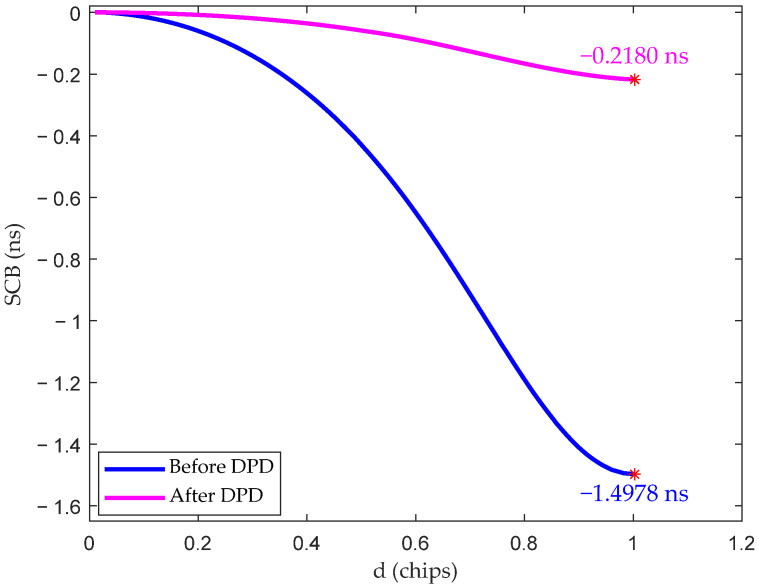
SCB experimental results of the proposed method. * marks the maximum value of the corresponding curve.

Based on the model shown in Figure 2, an experimental platform was established to verify the proposed method. The measurement results presented in Figure 10 indicate that, under the influence of the HPA, the proposed method is able to reduce the SCB from 1.4978 ns to 0.2180 ns. Overall, the experimental results are consistent with the simulation results in terms of performance trends, with a noticeable improvement in SCB after compensation.

A certain discrepancy can be observed between the experimental results and the simulation results in Section 4.3. The difference is mainly attributed to the fact that the AM-AM and AM-PM (amplitude modulation to phase modulation) characteristics cannot fully and accurately represent the complex nonlinear behavior of the practical PA, resulting in some distortion components remaining uncompensated.

## 5. Discussion

### 5.1. Discussion of Results

To achieve higher-precision positioning services, this paper investigates the distortion issues in the navigation signal transmission channel. We propose a joint compensation method of the HPA and filter in the navigation signal transmission channel. Selecting SCB as the evaluation metric, the proposed method is verified through simulation and experiments. The following conclusions can be drawn:

- The distortion caused by the post-filter leads to the asymmetry of the CCF, which affects the SCB result. However, after compensation by the predistortion filter, the SCB can be effectively compensated, reducing the pseudorange bias.
- The predistortion filter breaks the constant envelope characteristics of the signal, causing nonlinear distortion after the signal passing through the HPA, reducing the compensation performance of the predistortion filter.
- After joint compensation of the filter and the HPA, the SCB is significantly reduced and effectively controlled.

The research presented in this paper serves as a reference for optimizing the design of navigation satellite payloads and provides strong support for improving navigation signal quality and system reliability.

### 5.2. Limitations and Future Work

Although the proposed joint compensation method has achieved good results in compensating for signal distortions, several limitations should be acknowledged, which also indicate directions for future research.

The HPA model employed in this study is constructed based on memoryless models of AM-AM and AM-PM. However, for the signals with large bandwidths case, the PA may exhibit memory effects, which cannot be accurately captured by memoryless nonlinear models and may degrade the predistortion performance. Future work will incorporate memory effects of PAs.

## Figures and Tables

**Table 1:** Group delay error before and after optimization of predistortion filter (ns).

Group Delay Error	Maximum Error	Mean Error
Before Optimization	0.1158	0.04813
After Optimization	0.004565	0.001147

## Data Availability

The original contributions presented in this study are included in the article. Further inquiries can be directed to the corresponding authors.

## Abbreviations

The following abbreviations are used in this manuscript:

GNSS	Global Navigation Satellite System
PNT	Positioning, Navigation, and Timing
SCB	S-curve Bias
HPA	High-power Amplifier
PA	Power Amplifier
DPD	Digital Predistortion
QRD-RLS	QR-Decomposition Recursive Least Squares
LUT	Lookup Table
EVM	Error Vector Magnitude
CCF	Cross-correlation Function
EMLP	Early-Mines-Late Power
AM-AM	Amplitude Modulation to Amplitude Modulation
AM-PM	Amplitude Modulation to Phase Modulation
